# Implementation of the Canadian Emergency Department Triage and Acuity Scale (CTAS) in an Urgent Care Center in Saudi Arabia

**DOI:** 10.1186/s12245-016-0112-9

**Published:** 2016-06-10

**Authors:** Abdullah Arafat, Ali Al-Farhan, Hiba Abu Khalil

**Affiliations:** King Saud ben Abdulaziz University for Health Sciences, Riyadh, Saudi Arabia; King Abdulaziz Medical City National Guard, Riyadh, Saudi Arabia

**Keywords:** CTAS, Emergency, Saudi Arabia, Triage, Urgent care

## Abstract

**Objectives:**

The objectives of the study is to review and assess the implementation of the applied modified five-level triage and acuity scale triage system in AL-Yarmook Urgent Care Center (UCC), King Abdulaziz Residential City, Riyadh, Saudi Arabia.

**Method:**

An observational cross-sectional study was conducted, where a data collection sheet was designed and distributed to triage nurses. The data collection was done during the triage process and was directly observed by the co-investigator. The triage system was reviewed by measuring three time intervals as quality indicators: time before triage (TBT), time before being seen by physician (TBP), and total length of stay (TLS) taking in consideration the timing of presentation and the level of triage.

**Results:**

A total of 187 patients visiting the UCC during December 2014 were included. There was an almost equal distribution of males 98 (52 %) and females 89 (48 %) with most of the patients being in the age group of 14 years and younger (*n* = 85, 46 %). The visits of the patients were classified according to the level of triage from patients to be seen immediately by the physician to those who had been triaged out. Overall, 173 patients (92.5 %) were seen by the physician in a timely manner according to triage guidelines, while 14 patients (7.5 %) were not. The mean time was 5.36 min in TBT, 22.6 min in TBP, and 59 min in TLS. The median time to be seen by the physician was significantly greater (*p* = 0.001) for the urgent cases on the weekends (25 min; IQR, 21,30) as compared to the weekdays (17 min; IQR, 14,21). Generally, the results did not show significant increases in TBT, TBP, the number of patients not seen at the proper time, or referral and admission rates during weekends.

**Conclusion:**

The Canadian Emergency Department Triage and Acuity Scale (CTAS) is adaptable to countries beyond Canada and can be implemented successfully. The applied CTAS triage system in Al-Yarmouk UCC in Riyadh, Saudi Arabia, is considered to be well applied. Overall, urgent cases have been seen by physicians in a timely manner according to the triage system, and there was no delay in the management of critical cases which need prompt attention.

## Introduction

Emergency departments (ED) are the first recipient of emergency cases among healthcare facilities but the severity of these cases widely varies from severe life-threatening cases (such as Acute Coronary Syndromes) to mild ones (such as indigestion). Many studies have shown that 50 % of the ED visits are non-urgent cases; as a result, ED frequently become congested resulting in patients with urgent condition having delay in getting proper management [1, 2]. The goal of ED triage is to classify the patients into categories based on the urgency of their conditions so that every patient is managed in a timely manner.

The term “triage” is derived from the French word “trier”, which means to sort [3]. The first implementation of triage concept goes back to the nineteenth century when Baron Dominique-Jean Larry (1766–1842), Napoleon’s surgeon, had started to apply a classification system for wounds on the battlefield; his system was based on an essential concept which was treating and evacuating those requiring the most urgent medical attention, but the first use of the term triage was during World War 1 [4, 5]. With the growing demand to have a tool to deal with the overcrowding in ED, the term of triage in medicine has showed up, which is the process classifying the patient according to the urgency of their medical conditions regardless of any other factors such as religion, nationality, race, gender, age, socioeconomic status, time of arrival, and insurance. Any triage process is influenced by three factors: patients’ characteristics, triage decision maker (staff factors), and the healthcare setting. The interrelation between these factors is important to get an effective triage process [6].

The main goal of any triage system is to provide the initial assessment and the subsequent determining of patient’s category, the initiation of early intervention if necessary, and ensuring that every patient is seen by physician in timely manner. With the later development of modern healthcare systems, a lot of well-studied and validated triage systems have been applied. Some of the important scales that an influence on the modern triage systems are the Australian Triage Scale (ATS), Canadian Emergency Department Triage and Acuity Scale (CTAS), Manchester Triage Scale (MTS), and Emergency Severity index (ESI) [7–9].

In Saudi Arabia, the application of different triage systems is guided by a manual for organizing nursing services in the country with triage system guidelines which was issued by the Ministry of Health in 2003 [10]. The CTAS is widely implemented at tertiary centers and overall the country. Dr Robert Beveridge led the development of CTAS based on the older Australasian National Triage Scale [10]. The first CTAS guideline was published after years of work in 1999; since that time, it has been vastly reviewed, studied, and validated and a pediatric triage guidelines was published later in 2001 [10]. CTAS is a five-level triage system consisting of first level “Resuscitation” (to be seen immediately), second level “Emergent” (to be seen <15 min), third level “Urgent” (to be seen <30 min), fourth level “Less Urgent” (to be seen <60 min), and fifth level “Non-Urgent” (to be seen <120 min).

Al-Yarmook (UCC) is one of Saudi Arabian National Guard Health Affairs facilities that serve the residents of King Abdulaziz Residential City in Riyadh (70,000 population). A modified CTAS is implanted at this facility because it is an UCC unit; the system divided the patients into five levels which are level 1 (immediately), level 2 (<15 min), level 3 (<30 min), level 4 (<60 min), and level 5 (triaged out). The applied CTAS in Al Yarmook Urgent Care Center has some modification in the patients’ disposal. In which patient in level 5 (Non Urgent) are triaged away with an appointment to the available primary care clinics in their area while those patients have to be seen within 120 min according to the CTAS criteria.

The main goal of this study was to review the implementation of the applied modified CTAS triage system at Al-Yarmook UCC in King Abdulaziz Residential City, Riyadh, Saudi Arabia.

## Method

This is a cross-sectional survey to review the outcomes of triage system in Al-Yarmook UCC. It was done during the month of December 2014 and was ethically approved and funded by King Abdullah International Medical Research Center (KAIMRC) under research protocol (RC13/071).

This UCC is one of the National Guard Health Affairs health facilities with a capacity of six beds (four trauma beds and two short stay beds). There are three shifts for the physicians which are morning (7 a.m.–3 p.m.), evening (3 p.m.–11 p.m.), and night (11 p.m.–7 a.m.) with two physicians during the evening shift and one during the others. All triage nurses in the UCC (12 nurses) are trained to apply the CTAS by attending a course conducted in King Saud Ben AbdulAziz University for Health Sciences in Riyadh.

A data collection sheet was designed by the co-investigators and distributed to triage nurses. The sheet includes numerical and categorical variables (demographic variables, time interval-related variables). The data collection was done during the triage process and was directly observed by the co-investigator. According to the center charts, 1800 patients visited the center in September 2014. With a finite population size of 1800 (based on the center charts), assuming that 15 % of patients at all levels of triage are not seen in timely manner, we estimated a sample size of 175 at 95 % CI with a margin of error .05. This was adjusted up to 187 for possible data losses. Piface® was used to calculate the sample size with CI single proportion. We divided the patients into two groups (weekdays, weekends) and further divided into different day and time periods. All the patients who visited the UCC during the month of December 2014 were included in the study.

The data was collected in convenience sampling technique. In which any patient who visited the UCC during the co-investigators shifts had been included. With a sample size of 187, we divided the visits into weekdays (118) and weekends (69). The data was collected in 5 days weekly (three weekdays, two weekends) with equal distribution between every shift.

The data was entered and analyzed using SPSS software. Time interval was measured to assess the effectiveness of triage system which are time before triage (TBT), time to be seen by physician (TBP), and total length of stay (TLS).The mean, median, and SD of those intervals were measured. The patients were divided into six age groups, and the frequency of each group was presented in charts with the frequency of each gender. The patients were divided into four time categories; the frequency of each triage level was measured and compared with the time interval to determine if it meets the triage guidelines. The median TBP according to the standard level is less than 15 min for level 1/2 (Immediate/Emergent), less than 30 min for level 3 (Urgent), and less than 60 min for level 4 (Less urgent) cases was measured for different triage levels and visit times. The discharge time of the patients was captured using the UCC QuadraMed medical system. Also, a comparison was done between the weekday and weekend data to discover if there is a significant difference between both. A *p* value <0.05 is considered as significant.

## Result

The study included a total of 187 patients visiting the Urgent Care Center during the period of December 2014. Table 1 shows the breakdown of the selected patients by demographics and day/time of their visit. There was an almost equal distribution of males 98 (52 %) and females 89 (48 %) with most of the patients being in the age groups of 15 to <30 years (*n* = 54, 29 %) and 14 to >5 years (*n* = 50, 27 %). Almost all of the patients 173 (97 %) were residents of the residential city.

**Table 1:** Demographics and time of presentation to the Urgent Care Center

	Number	Percent (%)
Age groups		
Up to 1 year	13	7
>1 to 5 years	22	12
>5 to 14 years	50	27
15 to <30 years	54	29
30 to <50 years	28	15
50+ years	20	11
Gender		
Male	98	52
Female	89	48
Residence^a^		
In Iskan	173	93
Out of Iskan	6	3
Time of presentation		
8 a.m.–12 noon	21	11
12:01–4 p.m.	33	18
4:01–8 p.m.	88	47
8:01 p.m.–midnight	45	24

^a^Missing = 8 for residence

Almost half of the visits were in the evening shift of 4–8 p.m., i.e. 88 (47 %) patients, with another 45(24 %) patients from the night shift of 8 p.m.—midnight (Table 1). The sample included 118 (63 %) patient visits on weekdays and 69 (37 %) visits on weekends. The visit of the patients was classified according to the level of triage from patients to be seen immediately by the physician to those who had been triaged out as shown in Table 2. There were 23 (12 %) patients who were classified as immediate/urgent (level 1) by triage. There was an equal distribution for the other three triage levels with 50 (27 %) patients being triaged out. There was no significant difference (*p* = 0.16) in the distribution of level of triage over the weekdays/weekends.

**Table 2:** Frequency of triage level comparison by visit day (weekdays/weekends)

		Visit day		
Level of triage	All days	Weekdays	Weekends	*p* value
To be seen immediately/emergent (<15 min)	23	16	7	0.16
	(12 %)	(14 %)	(10 %)	
Urgent (<30 min)	59	43	16	
	(32 %)	(36 %)	(23 %)	
Less urgent (<1 h)	55	32	23	
	(29 %)	(27 %)	(33 %)	
Non-urgent (triaged out)	50	27	23	
	(27 %)	(23 %)	(33 %)	
Total	187	118	69	
	(100 %)	(100 %)	(100 %)	

The waiting time for the patients to be seen at each triage level by the triage nurse and physicians, as well as the total time for disposal was compared between the weekday and weekend visits. The distribution of time was skewed to the right so the Mann-Whitney *U* test was used to compare the median times as shown in Table 3. There was generally no difference in the time to be seen by triage nurse (TBT) by visit day as shown in Table 3. The median TBP was significantly greater (*p* = 0.001) for the urgent cases on the weekends (25 min; IQR, 21,30) as compared to the weekdays (17 min; IQR, 14,21). There was a borderline difference (*p* = 0.08) for the non-urgent cases as well with the weekend median time of 35 min (IQR, 25,43) being slightly more than the 30-min (IQR, 20,36) time on weekdays. The triaged out cases were not included in this part because they did not require to be seen by a physician. Table 3 also shows that there was no significant difference in the overall median time from patient registration to disposal over the weekdays versus weekends.

**Table 3:** Waiting times for patients to be seen by triage nurse, physician, and total time to disposal

	Weekdays		Weekends		
	*N*	Median (IQR)	*N*	Median (IQR)	*p* value
Time to be seen by triage nurse (TBT)					
Immediate/emergent (<15 min)	16	1.0 (1.0, 2.0)	7	1.0 (0.0, 2.0)	>0.99
Urgent (<30 min)	43	5.0 (3.0, 6.0)	16	5.0 (3.0, 11.0)	0.88
Less urgent (<1 h)	32	5.0 (4.0, 9.75)	23	6.0 (3.0, 8.0)	0.91
Non-urgent (triaged out)	27	6.0 (4.0, 7.0)	23	4.0 (3.0, 8.0)	0.54
Time to be seen by physician (TBP)					
Immediate/emergent (<15 min)	16	3.0 (2.0, 7.0)	6	7.5 (2.5, 9.0)	0.18
Urgent (<30 min)	43	17.0 (14.0, 21.0)	16	25.0 (21.0, 30.0)	0.001
Less urgent (<1 h)	32	30.0 (20.0, 35.75)	23	35.0 (25.0, 43.0)	0.08
Total length of stay (TLS)					
Immediate/emergent (<15 min)	16	93.0 (53.25,245.75)	7	83.0 (38.0, 165.0)	>0.99
Urgent (<30 min)	43	61.0 (35.0, 80.0)	16	46.5 (41.5, 91.25)	0.83
Less urgent (<1 h)	31	52.0 (35.0, 64.0)	23	53.0 (40.0, 61.0)	1.00
Non-urgent	24	13.0 (10.25, 15.0)	23	10.0 (9.0, 15.0)	0.31

**Fig. 1:**
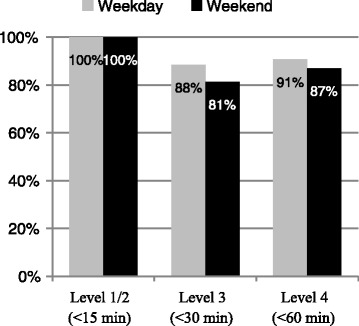
Visit that met the triage criteria

All the level 1/2 cases were seen within the required time on both visit days. The majority of cases (>80 %) were also seen within the required time for both levels 3 and 4 as shown in Fig. 1. There was no significant difference in the proportion of patients seen on time over weekdays versus weekends.

## Discussion

The implemented triage system in AL-Yarmook UCC is a modified form of the original CTAS. The CTAS was developed in the late 1990s and was extensively validated since that time [7–9]. As far as we know, there has been no previous similar study that was conducted in this institution. The primary objectives of any triage system are to ensure that patients are classified according to the severity of their conditions and being managed in a timely manner. Our data assessed the effectiveness and performance of the applied triage system by considering the time interval (TBT, TBP, TLS) as quality indicators.

The majority of our UCC patients were at level 3 (59, 32 %), which is not similar to the result of applied CTAS among Andorra population which showed a majority of level 4 (47 %) [2]. The median values of TBT for all triage levels are similar to the result of a previous study that was conducted in King Faisal specialist Hospital and Research Center, Saudi Arabia [11]. The median values for (TLS) is extremely less than that of both previous studies, and this can be explained by the limited admission ability for our UCC and the available referral to the near to King Abdulaziz Medical City ED [2, 11].

The median waiting TBP for both weekday and weekend visits was 32 and 23 min, respectively, which was significantly less than a previous study reviewing CTAS in a hospital in Riyadh [11].

A valid triage system requires that every patient should be reassessed if the waiting time exceeded the triage guidelines. The reassessment was not applied in AL-Yarmook UCC, and we recommend that this has to be reviewed with the appropriate authority. Also, the patient’s waiting area was out of the nurses’ sight which may limit a direct observation of patients by nurses while waiting. Also, it is clearly shown from the result that there is a misunderstanding in the health-seeking behavior of our population with this high number of triaged out patients, 50 (27 %). This high number of triaged out patients can be explained by the availability of the primary healthcare services in the residential city. Since that King Abdulaziz Residential City closed community with good primary healthcare services, we have the duty to raise the awareness of the residential population about the proper healthcare-seeking behavior.

Overall, urgent cases have been seen by physicians in a timely manner according to the triage system, and there was no delay in the management of critical cases which need prompt attention.

The overall referral rate in our study was 13.7 % which is similar to the previous two studies were conducted in Riyadh, Saudi Arabia [11, 12]. Half of them had been referred to King Abdulaziz Medical City ED, and the other half were referred to the primary healthcare satellite clinic with no significant change in the referral rate on weekends.

## Conclusion

With considering the time intervals to meet the triage system guidelines as quality indicators, the applied triage system in Al-Yarmook UCC is considered to be well applied. Also, we think that it is better to have a waiting area for both genders that can be directly observed by triage nurse, and the concept of reassessment of the patients’ condition after spending more than proper waiting time has to be emphasized by the triage nurse.
